# Essential Role of Potassium in Apple and Its Implications for Management of Orchard Fertilization

**DOI:** 10.3390/plants10122624

**Published:** 2021-11-29

**Authors:** Andrei Kuzin, Alexei Solovchenko

**Affiliations:** 1 I.V. Michurin Federal Scientific Center, 393774 Michurinsk, Russia; solovchenko@mail.bio.msu.ru; 2 Faculty of Biology, Lomonosov Moscow State University, 119234 Moscow, Russia; 3 Institute of Natural Sciences, G.R. Derzhavin Tambov State University, 392000 Tambov, Russia

**Keywords:** potassium, apple tree, fruit, management of orchard fertilization, mineral nutrition, fertigation, pedoclimatic characterization, microbial cultures, crop load, image processing, K solubilizing bacteria

## Abstract

K (K) is of paramount importance for apple (*Malus × domestica* Borkh.), not only for tree growth and development but also for the size and quality of fruit yield. The apple plant’s demand for K varies, along with the progression of phenological phases, during the growing season. The K demand peaks during ripening of fruits featuring relatively high concentration of K comparable to that of the leaves. The mainstream method of apple tree K fertilization is through application of the fertilizer to the soils to improve K uptake by the roots. The bioavailability of K depends on assorted various factors, including pH, interaction with other nutrients in soil solution, temperature, and humidity. An important role in making the K from soil available for uptake by plants is played by plant growth-promoting microorganisms (PGPM), and the specific role of the PGPM is discussed. Advantages of fertigation (the combination of irrigation and fertilization) as an approach include allowing to balance application rate of K fertilizer against its variable demand by plants during the growing season. Excess K in the soil leads to competitive inhibition of calcium uptake by plants. The K-dependent deficiency of Ca leads to its predominant channeling to the leaves and hence to its decline in fruits. Consequently, the apple fruits affected by the K/Ca imbalance frequently develop physiological disorders in storage. This emphasizes the importance of the balanced K application, especially during the last months of the growing season, depending on the crop load and the actual K demand. The potential use of modern approaches to automated crop load estimation through machine vision for adjustment of K fertilization is underlined.

## 1. Introduction

Potassium (K) is among essential mineral nutrients with long-known importance for plant development and yields. Its content in plant ash can be as high as 50%, so K attracts close attention of plant physiologists and practitioners to develop recommendations for K-fertilizing of apple (*Malus* × *domestica* Borkh.) fruit plants to achieve their best performance in the field [1]. A significant body of evidence on the important role of K in plant organism has been accumulated over last 100 years [2]. K is the main cationic inorganic nutrient for plants, and its special role is largely determined by its presence in plants in the form of a simple free cation K^+^ [3]. 

As such, K is directly involved in vital functions of plant organism, including the regulation of photosynthesis [4], osmotic regulation of stomata activity and transpiration [5,6], growth and development [7], and responses to abiotic stresses [8], e.g., salinity stress [9]. A sufficient supply of K is crucial for cell membrane stability, root growth, leaf expansion, and dry mass accumulation in plants. In the plants dwelling in arid regions, K also augments water uptake and conservation, thereby reducing drought stress [10]. This nutrient also participates in the formation of the resilience to the low-temperature stress [11] and to biotic stresses caused by fungal, bacterial, and viral agents, as well y insects and nematodes [12,13]. Optimal potassium supply is significant for the shoot and root growth and development (Table 1).

Since K is involved in the regulation of plant homeostasis [14], its optimal supply is very important under the conditions of climate change and in view of the need to obtain good yields of high-quality fruits. Reaching this goal requires up-to-date fertilization management systems presuming balanced use of both organic and chemical fertilizers [15]. Naturally, sufficient K supply is not the sole determinant of stress resilience, but apple plants frequently encounter problems with K acquisition, leading to a decline in fruit yield and quality [16,17]. Notably, an adequate supply of K is essential for uptake of other nutrients, particularly nitrogen [18,19]. K is an important constituent of apple fruits. Its dry mass percentage depends on varieties, typically ranging from 0.6% to 1.1%, which is commensurate to the foliar K content [20,21,22].

The adequate supply of K is crucial for the productivity of orchards: its beneficial effects on yield of young trees reaches 12% and 20% older trees (Table 2) [23]. The main types of K fertilizers are applied through fertigation by using various sources of K salts, such as potassium chloride (KCl), potassium sulfate (K_2_SO_4_), potassium nitrate (KNO_3_), and mono-potassium phosphate (KH_2_PO_4_). Less common K fertilizers are potassium thiosulfate (K_2_S_2_O_3_) and potassium carbonate (K_2_CO_3_) [24]. The current situation with potassium fertilizer application, their description, and perspectives are well presented in review Zörb et al. [25]. 

In this review, we generalized on the role of K in apple tree nutrition and the yield formation. We highlighted the novel trends of the use of microbial biofertilizers to reduce the impact on the environment and pointed out the promise of on-line adjustment of K application rate during the growing season to avoid over-fertilizing employing automated crop load assessed via machine vision.

## 2. The Coverage and the Focus of the Review

As noted above, we aimed at highlighting the key problems associated with K nutrition of apple trees, together with the traditional and alternative approaches to its optimization. This discussion is based on the basic determinants of K availability to plants. During preparation of this review, we have screened more than 600 articles with the relevant keywords (acidity, apple, availability, bacteria, biofertilizers, calcium, cation, concentration, copper, counting, crop load, fertigation, fruit, fungi, growth, horticulture, humidity, interaction, ion, irrigation, leaf, magnesium, microorganisms, mineral nutrition, nitration, nitrogen, phenology, phosphorus, plant physiology, potassium, precision, root, sodium, soil, solubilization, stage, status, sulfur, temperature, transpiration, transport, uptake, yield, etc.). After final selection, the shortlist of more than 200 contributions of the authors working on this topic for many years was produced. This list was further refined with the topics of K application rate correction, also using the modern approaches to crop load assessment [26].

## 3. Seasonal and Developmental Variation of K Demand in Apple

### 3.1. Bud Break—Full Bloom (Phases 07–65 BBCH Scale)

The nutrient demand of apple plants varies at different growth stages [27]. The data in Table 3 show that, despite the various potassium supply, the leaf potassium status of two different apple cultivars changed uniformly through the growing season (Table 3). Foliar K concentration at bloom (38 days after bud break, DABB) was 2.3% (here and below, dry mass percentages are reported) in Gala/M26 plants in a sand pot culture [28]. Foliar K content in the apple varieties Ligol, Venyaminovskoe, and Bogatyr grafted on B396 rootstock in three orchards in Central Russia varied in the range 0.6–1.1% (10 DABB; the Mouse Ear stage or 53–54 according to the BBCH scale (this scale is used to identify the phenological development stages of plants; its abbreviation derives from the names of the originally participating stakeholders: “Biologische Bundesanstalt, Bundessortenamt und CHemische Industrie”) [29]. The foliar K concentration increased for a few days (16 DABB, 57 BBCH) to 1.1–1.5% by the time of bloom (40 DABB, 65 BBCH) and then decreased again to ca. 1.1%.

In apple, bud break and the initial shoot growth occur in early spring at the expense of internal reserves of mineral and organic nutrients formed in the previous season. The K content in the one-year-old apple (Sinap Orlovsky) shoots and in March before budbreak was 0.36–0.45% [30]. This can explain a depletion of foliar K at 10 DABB. It gets replenished later when the K taken up by the roots reaches the aboveground plant organs. Then, with acceleration of the metabolism during flowering, foliar K levels declined again. Hence, the demand for K increases at the phases 31–51 (shoot growth start–generative bud swelling).

The soil temperature should also be taken into account: when it is below 15 °C, the shoot growth rate and the rate of photosynthesis are low [31]. Accordingly, the activity of the root system and the uptake of nutrients, including K, are also slow [32,33,34,35]. In this case, it is better to switch from soil K fertilization to foliar application of K-containing formulations. An increase in K supply during this period will augment the flower opening and eventually improve the fruit set. Still, the problem of optimal balance of forms and methods of K supplementations across the growing season requires further studies.

### 3.2. Full Bloom—40-mm Fruit (65–74 BBCH Scale)

**Figure 1:**
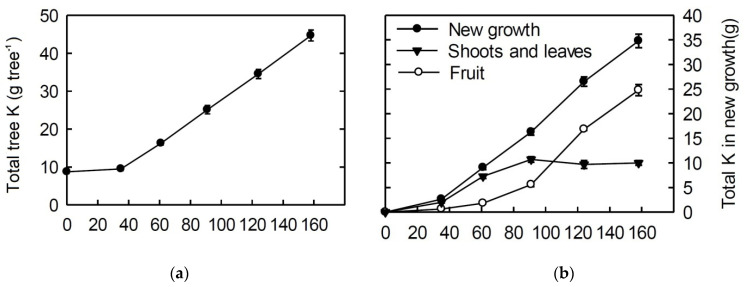
Total K accumulation in the entire tree (**a**) and in new growth (**b**) of 6-year-old ‘Gala’/M.26 trees grown in sand culture under a complete nutrient supply regime. The six points correspond with bud break, bloom, end of spur leaf growth, end of shoot growth, rapid fruit expansion period, and fruit harvest, respectively [28].

During this period, the K demand increases because of the intensive growth of shoots and fruitlets. As a result, the total K content in plants increases linearly to the level twice the initial K concentration (Figure 1; also see Reference [36]). 

Fruits K demand is relatively low at this period when K is spent mainly on the growth of leaves and shoots. Therefore, the foliar K concentration of apple trees in this period is less cultivar-specific; it depends more on the intensity of growth (and, hence, on the age of orchard), the concentration of exchangeable soil K, and the weather. Previously, we reported that the foliar K decreased from 2.0% to 1.7% [21] or remained almost unchanged at the level of 1.5% to 2.0% in apple trees of different ages [24].

As a rule, leaf sampling for assessment of plan K status is recommended at 60–80 DAFB [37]. Earlier sampling is proposed to optimize the K fertigation—14 DAFB and two weeks later [38]. In our previous study, foliar K in the leaves sampled immediately after flowering displayed the closest correlation with the yield [21]. Overall, the developmental variation of adequate K supply to apple plants and the algorithms of the adjustment of K application rates, depending on various environmental, physiological, and technological factors, has not been sufficiently studied. 

### 3.3. 40-mm Fruit—Picking Maturity (74–87 BBCH Scale)

**Figure 2:**
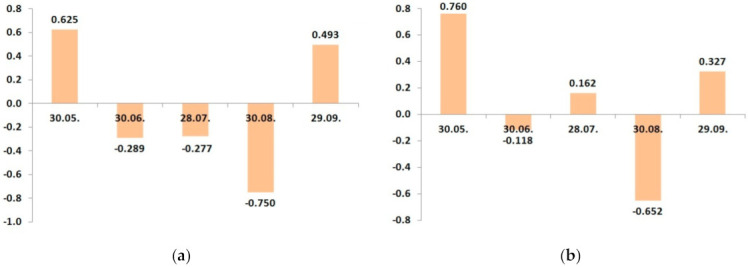
Dynamic changes of correlation coefficients between the potassium leaf status and yield cv. ‘Zhigulevskoye’ in growing seasons: (**a**) 2016; (**b**) 2017 [22].

These stages are characterized by fruits growth and development. Consequently, the fruits constitute the main sink of the K taken up at that time. For example, Cheng [28] reports that the K content in 6-year-old Gala/M26 trees at the time of bud break was 8.7 g per tree, whereas, after leaf fall, it was 12.2 g per tree. By contrast, at harvest, it was 44.7 g per tree, including 24.8 g in the fruits. Even such a brief consideration makes clear the paramount role of K supply during this period for high yield, so it has been a focus of research for many years. In this period, the leaf potassium concentration decreases, while there is a strong need for fruits in this nutrient (Figure 2). Thus, an increase in the frequency of fertilization from two to four times (the last two applications were two and three months after bloom, respectively) lead to a significant increase in the yield [39].

However, it is essential to also choose the optimal fertilizer application rate in this period to increase the yield [40]. For example, applying K fertilizers in July and August (combined with nitrogen and phosphorus applications) significantly increased the yield of five apple cultivars in British Columbia [41]. At the same time, the lack of K leads to a significant decrease in fruit size and, hence, yield [42,43,44,45].

The optimal K supply in this period is also important for the increase of the fruit quality [46]. Thus, K fertilization positively affected fruit color, total soluble solids (TSS) content, and the pulp density [47]. At the same time, excessive application of K can lead to a decrease in the fruit calcium concentration [48,49]. For example, K levels above 11.7 mg kg^−1^ in the fruits of Gala/M26 apple cultivar led to a decrease in their calcium content [17]. Thus, one of the criteria of the optimal K supply is the absence of its negative effect on the fruit calcium levels, which is crucial for the fruit storability. 

## 4. Uptake of K by Apple Trees

### 4.1. Influence of Soil Acidity (Soil pH)

**Figure 3:**
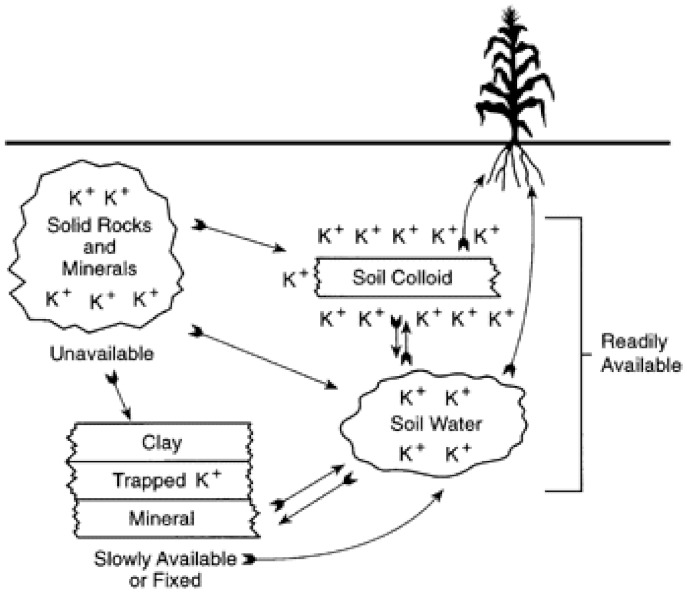
Relationship among different form of potassium in the soil-plant system [50].

Soil K existing in three forms—unavailable, slowly available, and readily available (Figure 3). The unavailable K comprises up to 98% of the total soil K [50]. In effect, plants can take up only about 1% of soil K; therefore, K fertilizing is very important. 

Plant roots absorb the bulk of K, and then it rises, in a dissolved form, in xylem sap to aboveground organs [51]. From the soil solution, K^+^ is absorbed by the epidermal and cortical cells of the roots. As soon as K^+^ is inside the root symplast, it can be stored in vacuoles, performs osmotic functions, or transported to the shoot through the xylem [52]. On their way from the roots to the organs of consumption, ions of K pass through cell membranes via several K^+^ transport systems [53]. At high concentrations of exchangeable soil K^+^, ionic forms of the nutrient get inside the roots through the cell membrane via channels formed by carrier proteins augmenting the ion uptake. At low concentrations of soil K, activation of active transport systems is necessary to absorb K^+^ against the electrochemical gradient [53].

Uptake of K in higher plants occurs via a typical dual-affinity (high and low) mechanism operating at different external K concentrations switching its modes depending on the external K^+^ availability [54]. Active K transport is normally engaged at a low external concentration of K^+^ (below 0.2 mmol L^−1^) affected the by K^+^/H^+^ exchanger pump. The low-affinity mechanism works predominantly when the external K^+^ concentration is higher than 1.0 mmol·L^−1^, and it is mainly affected by K^+^-channels [55]. The genes encoding the K^+^ carrier proteins and channels, e.g., KUP/HAK/KT, HKT, NHX, and CHX, were mapped and characterized [56]. As a result, the K influx depends on the amount and activity of carrier proteins, transpiration, and other factors [57], so it is necessary to create an optimal concentration of K in the soil to facilitate its absorption into plants.

Since the structure and activity of carrier proteins for various nutrients is genetically programmed, different apple rootstocks display different pH tolerance. Generally, foliar levels of calcium, phosphorus, and molybdenum increase with an increase in the soil pH, whereas the absorption of iron, manganese, and nickel decreased, and the adsorption of copper, sodium, zinc, magnesium, sulfur, and K remained approximately at the same level [58]. Valverdi at al. [59] reported a significant variation of K, iron, and molybdenum uptake in the apple rootstocks G890, G41, M9, and B9 in response to the soil acidity.

Overapplication of many types of fertilizers causes an increase in soil acidity. For example, an increase in nitrogen and phosphorus application rate and in the concentration of bioavailable phosphorus in the soil solution led to a pH decline from 5.8 to 4.9. Foliar K declines linearly with soil pH decline [60,61]. The increase in soil acidity results in a reduction of nutrient uptake, inhibition of plant growth, and deterioration of apple yield and quality (color, size, and sugar content) [62]. Overall, the problem of the dependence of K uptake and, hence, plant performance on soil pH still requires close attention.

### 4.2. Interactive Effects of Ions in the Soil on K Uptake

Much attention was paid by the researcher community to the interaction of nutrients in soil solution. In particular, K^+^ ions affect significantly the absorption and utilization of nitrogen and phosphorus from the fertilizers applied to the soil [63]. The interaction between nitrogen and K in the soil, as a rule, is beneficial for plants [63,64] since nitrate ions are co-transported with K^+^ [64,65,66,67].

The interaction between K and phosphorus also positively affected plant growth [64]. However, an increase in phosphorus application rate may reduce the availability of zinc and molybdenum in the soil [68]. Furthermore, high K application rates can also limit the uptake of calcium and magnesium by plants [22,64,69]. The application of K-magnesium fertilizers reduced the concentration of exchangeable soil calcium, so the foliar magnesium concentration did not increase after the fertilization [70]. 

In view of the above, the efficiency of K fertilizers and optimal application rate are the most topical problems [71] since the growers tend to overfertilize with K disregarding the local conditions [69]. To establish the optimal supply rate of plants with K, it is necessary to consider the conditions of a particular orchard: the soil pH, actual soil content, and availability of K and other nutrients. 

### 4.3. Soil Temperature and Humidity Effects on K Uptake

The uptake of nutrients is determined by the growth of roots towards the location of nutrients and the movement of nutrients to the site of their uptake [72]. The activity of the root system regarding nutrient uptake is highly dependent on the temperature of the soil [73]. The activity of soil microorganisms also plays a significant role in increasing the availability of the nutrient ions [74], including K^+^. Soil temperature affects the viscosity of the soil solution, which determines the mobility of ions dissolved in the soil solution [75,76]. Low soil temperature slows down root growth, along with physiological processes occurring in them, due to a decrease in hormonal activity [77] or hydraulic conductivity of cells [78], as well as due to a displacement of the carbohydrate balance in the roots [79].

Soil K exists in the following forms: dissolved, exchangeable, non-exchangeable or fixed, and as a composition of soil minerals [80]. The release of fixed or structural K from clay particles is the dominant mechanism of K release in agricultural soils of temperate climates [81]. The release of K from clay particles is an essential nutrient source for crop plants, and it depends on the soil structure and clay particles [82]. A decrease in soil moisture leads to strong adsorption of K ions to the clay particles surrounding the roots preventing uptake of K [83], and vice versa, the mobility of K and its uptake by plants from the soil increases with an increase in soil humidity [84,85,86].

### 4.4. The Application of Microbial Cultures for Soil K Mobilization

The key drivers for replace, at least partially, the chemical fertilizers (including K fertilizers) with organic fertilizers are mitigation of soil degradation and conservation of its microbiome and other aspects of soil health [87,88]. Thus, application of mineral fertilizers, especially nitrogen and K fertilizers, increases soil acidity [89,90]. This problem is relevant for many regions with slightly acidic soils, including Central Russia. There are several approaches to reducing soil acidity, particularly liming [90]. Recently, much attention has been paid to the use of biochar [91]. However, it is better to prevent an increase in soil acidity to critical values than to fight the soil acidification when it already happened. A possible proactive approach presumes a (partial) substitution of the mineral fertilizer application by application of biofertilizer, including microbial cultures. The microbial cultures also have the ability of potassium solubilization from insoluble potassium [92].

Many species of microorganisms dwelling in the rhizosphere of plants form symbioses with plants whom they supply with mobilized nutrients, as well as organic exometabolites with hormone-like properties [93,94]. Thus, the K-solubilizing microbes, including the bacteria (*Bacillus mucilaginosus*, *B. edaphicus*, *B. circulans*, *Acidithiobacillus ferrooxidans*, and *Paenibacillus* spp.) and fungi (*Aspergillus* spp. and *A. terreus*), make the insoluble soil K bioavailable for plants [95]. The bacteria, mostly from the genus *Bacillus*, can liberate K, silicon, and aluminum from insoluble minerals [96,97].

Normally, only 1–2% of the total soil K is bioavailable to plants through the soil solution, but the microorganisms from the rhizosphere significantly improve the K supply to plants [98,99]. Therefore, biofertilizers often improve growth, development, and fruit bearing of apple [100]. However, the use of *B. mucilaginosus* in an apple orchard on chernozem soils did not increase significantly the exchangeable K concentration in the rhizosphere of the trees; on the contrary, it was considerably lower than when mineral K fertilizer was applied [22]. Microbiological biofertilizers significantly affected apple yield only in combination with mineral K fertilizer application, although the latter were applied at a reduced rate [101,102]. Holb et al. [103] compared the nutrient uptake among 39 apple cultivars in non-irrigated conventional and organic orchards at soil pH 5.1. The application of mineral K fertilizers leads to a higher leaf nutrient concentration than organic fertilizer. Overall, it is not yet possible to replace completely the mineral fertilizers with microbial biofertilizers, although partial substitution seems to be quite possible. This would reduce the application of chemical fertilizers, thereby reducing the impact on the environment.

Certain bacterial strains can lower the pH of the cultivation medium facilitating the mobilization of phosphorus, which was otherwise scarcely available [104]. The use of microorganisms to increase nutrient availability in the rhizosphere and to optimize other aspects of soil fertility became a promising direction in the development of green agriculture. Plant growth-promoting microorganisms (PGPM) can be a potent tool for overcoming stresses caused by excessive acidity or alkalinity of the soil [105]. Some microorganisms have developed efficient mechanisms to mitigate extreme pH, e.g., by the synthesis of extracellular polysaccharides (in *Rhizobia* [106]) or glutathione tripeptide (in *Rhizobium tropici* bacteria demonstrating a highly acidic pH [107]).

There were also reports evidence that soil microorganisms can reduce the toxicity of excessive concentrations of ions (for example, aluminum) arising due to high acidity of soil [108]. Currently, little is known about soil pH regulation using PGPM, but the PGPM-based methods of the adjustment of soil acidity according to plants’ needs might be among the promising approaches of the future.

## 5. Approaches to Automated Precision Adjustment of K Application Rate

In a previous study, Wargo, with coworkers [109], verified fruit size is influenced more by crop load than by the amount of N applied to the apple tree. Later, the results of this study were confirmed in terms of nitrogen fertilizers by Nava and Dechen [110]. These authors proved that fruit size was consistently increased by K fertilization. The concentration of exchangeable K in apple orchard soil can change significantly during the growing season, depending on plant K demand and climate [22]. Fertigation and drip irrigation can substantially change the distribution of exchangeable K in different soil layers [111,112], especially to cover the peak K demand during fruit swelling and ripening [22,36]. Improper nutrient supply can induce or exacerbate alternate bearing typical of apple fruit crop [113,114,115,116,117]. Currently, the most common method for the control of alternate bearing is thinning of flowers and small fruits [116,117,118,119]. Numerous factors in control of floral bud set are involved in the development of alternate bearing, both external (light, temperature, moisture) and internal (nitrogen-to-carbon ratio, hormonal balance) factors [120]. The apple fruit growers can hardly manage such a complex combination of factors during the growing season and throughout the life cycle of orchards. A more simplistic but, at the same time, more manageable is based on control of the K supply and crop load. 

As it mentioned above, crop load largely determines the need for K in the second half of the growing season [121]. On the other hand, excessive soil K can suppress the uptake of calcium impairing its storability [122]. Therefore, it is very important to keep the K application in balance with its actual demand, which is expected to be proportional to the crop load. Accordingly, an important metric for the precise control of K application, e.g., via fertigation, would be the size of crop load [22,36], in combination with the output of traditional “wet” methods of the assay of soil K availability [123].

**Figure 4:**
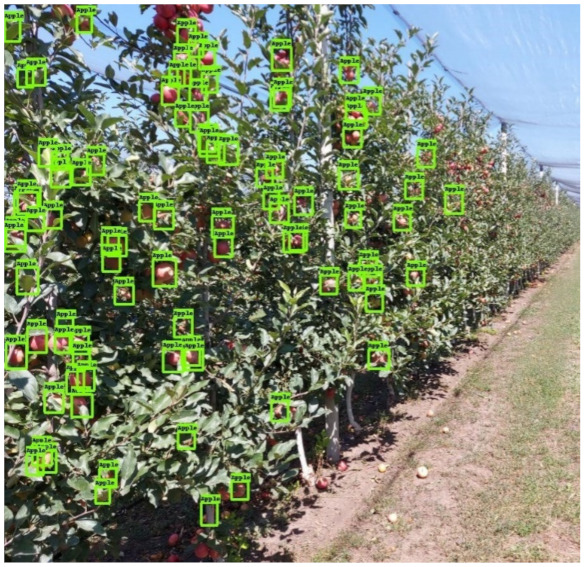
Automated apple fruit counting on images for crop load estimation in an industrial orchard (Solovchenko et al., unpublished).

The traditional method of apple crop load assessment by manual counting with subsequent interpolation is laborious, imprecise, and hardly feasible in large orchards. Currently, new approaches are being developed and implemented in framework of precision agriculture [124,125]. Those include automated crop load assessment techniques based on computer vision and machine learning (see, e.g., Figure 4), which are becoming [126,127,128] widespread and commercially available to fruit growers. These approaches provide the spatially resolved information on fruit density per unit area or even per single tree for the entire orchard. A computer management system processes the output of such systems to issue the control signal for variable K application via fertigation system of the orchard with high precision, both in time and space.

## 6. Conclusions and Future Research Directions

Admittedly, K is among the key nutrients for fruit crops, including apple. The K demand of apple tree varies throughout the growing season peaking during fruit expansion and ripening. Inter-season variation also takes place because of orchard aging and climatic fluctuations. The bioavailability of K for the uptake by plant roots depends on environmental factors, interaction with other nutrient ions, and soil microbiota. Oversupply of K suppresses the uptake of other nutrients, e.g., calcium, deteriorating the quality and storability of fruits. The actual K demand is proportional to crop load, so the K fertilization should be adjusted to match the actual K demand, which is a challenging task.

Currently, fertigation is the most effective and widespread technique for variable-rate K supplementation of apple trees with the necessary nutrients and water. Since many factors affect soil K availability and its uptake by the plants, it is hard to develop a one size-fits-all approach to K fertilization in apple orchards. New approaches developed in framework of precise horticulture allow the use of precise automated estimations of the apple crop load to control the K application via fertigation. The automated assessments and models still need validation against actual K levels in soil plants made with traditional “wet” methods and the correction for climate and soil conditions of specific orchards. The automated crop load estimation id is critical for expected yield estimation and, because of this, is essential for such organizational aspects as preparation of a needed number of workers for fruit harvesting, containers, and fruit storage facilities.

To conclude, the future research avenues would include the investigation of the interactive effects of K on soil microbiome, direct and indirect of K supply on stress resilience in fruit crops as a function of different systems of orchard management. Special attention should be paid to the development of techniques for automated non-invasive assessment of plant K nutrition status. Finally, the use of microbial biofertilizers containing the K-mobilizing microorganisms is expected to reduce the application rate of mineral K fertilizers, thereby reducing the pressure on the agricultural ecosystem of industrial orchard.

## Figures and Tables

**Table 1:** Developmental effects of different K supply on M9T337 [19]. Different letters denote significantly different values.

K Supply (mM K^+^)	Shoot Dry Weight (g plant ^−1^)	Root Dry Weight (g)	Root/Shoot Ratio
0	3.93 ± 0.14 ^d^	1.45 ± 0.08 ^d^	0.37 ± 0.03 ^c^
3	4.30 ± 0.16 ^c^	1.77 ± 0.09 ^c^	0.41 ± 0.03 ^ab^
6	5.37 ± 0.15 ^a^	2.30 ± 0.13 ^a^	0.43 ± 0.01 ^a^
9	4.84 ± 0.15 ^b^	1.94 ± 0.07 ^b^	0.40 ± 0.00 ^ab^
12	4.34 ± 0.27 ^c^	1.66 ± 0.10 ^c^	0.38 ± 0.01 ^bc^

**Table 2:** Potassium fertigation affects fruit yield of 3- and 4-year old apple trees on dwarf rootstock [23].

K_2_O Rate oz. tree^−1^ year	3-Year Old Trees		4-Year Old Trees	
	Yield, lb tree^−1^	Mean Fruit Weight, oz.	Yield, lb tree^−1^	Mean Fruit Weight, oz.
0	7.5	7.4	9.9	6.3
0.6	8.4	7.8	11.9	6.7

**Table 3:** Seasonal changes of leaf potassium content, % d.m. [22].

Date	Control N_20_P_15_	N_20_P_15_K_25_	N_20_P_15_K_35_	N_20_P_15_K_45_	N_20_P_15_K_20_	N_20_P_15_K_25_	N_20_P_15_K_30_	LSD_05_
‘Zhigulevskoye’								
30.05	1.81	1.89	1.74	1.97	1.76	1.92	1.73	0.10
30.06	1.61	1.21	1.29	1.56	1.28	1.21	1.26	0.08
28.07	1.36	1.09	1.32	1.33	1.33	1.29	1.24	0.08
30.08	1.22	1.05	1.00	0.98	0.94	0.88	0.98	0.06
29.09	1.30	1.27	1.31	1.52	1.12	1.37	1.44	0.08
‘Lobo’								
30.05	1.67	1.77	1.78	1.95	1.71	1.73	1.68	0.11
30.06	1.54	1.65	1.77	1.67	1.63	1.68	1.61	0.10
28.07	1.12	1.34	1.42	1.24	1.42	1.51	1.30	0.08
30.08	1.04	1.07	1.03	1.13	0.85	0.88	0.79	0.05
29.09	1.15	0.91	1.09	1.03	0.85	0.87	0.96	0.06
